# D-galactose-induced osteoporosis in rodents—Model development, skeletal characteristics, and optimal dosing regimens: A scoping review

**DOI:** 10.17305/bb.2026.13914

**Published:** 2026-03-17

**Authors:** Yuanzhong Wang, Li Lian Goh, Kok-Yong Chin

**Affiliations:** 1 Department of Pharmacology, Faculty of Medicine, Universiti Kebangsaan Malaysia, Cheras, Malaysia; 2 Chongqing Chemical Industry Vocational College, Changshou, China; 3 School of Medicine, IMU University, Bukit Jalil, Malaysia

**Keywords:** Ageing, bone, osteopenia, senescence, skeleton

## Abstract

D-galactose has been extensively utilized as a compound to accelerate aging and induce osteoporosis in rodent models. However, variations in dosage, administration routes, and experimental protocols have resulted in inconsistent findings. This scoping review aims to compile preclinical evidence on the use of D-galactose to induce bone loss, with a particular focus on identifying optimal dosing regimens and their specific impacts on skeletal indices. A systematic literature search was conducted in PubMed, Scopus, and Web of Science using predefined terms related to D-galactose-induced osteoporosis in rodents. Eligible studies were selected based on inclusion criteria, concentrating on both *in vivo* and *in vitro* models. A total of 44 studies met these criteria, primarily involving rats and mice. D-galactose was administered via subcutaneous, intraperitoneal, or oral routes, with doses ranging from 10 to 10,000 mg/kg/day for durations spanning 20 days to 5 months. The most commonly employed regimen was 200 mg/kg/day for 8 weeks. D-galactose consistently induced osteoporotic changes, including reduced bone mineral density, compromised microarchitecture, decreased biomechanical strength, and alterations in bone turnover markers. These skeletal changes were associated with oxidative stress, cellular senescence, and ferroptosis. Susceptibility to these effects was influenced by sex, species, and age, with male rodents generally exhibiting greater sensitivity. In conclusion, D-galactose serves as an effective model for age-related osteoporosis in rodents, primarily mediated by oxidative stress and accelerated cellular senescence. A regimen of 200 mg/kg/day administered subcutaneously or intraperitoneally for 8 weeks is commonly used to replicate osteoporotic phenotypes. Greater standardization of protocols is necessary to enhance reproducibility and translational relevance in future studies.

## Introduction

Osteoporosis is a chronic skeletal disorder characterized by impaired bone quality and disrupted structural integrity, predominantly affecting individuals over the age of 50, with women being disproportionately impacted [1, 2]. This condition is defined by reduced bone mineral density and deterioration of bone microarchitecture, both of which increase the risk of fragility fractures and functional decline [3]. Often progressing silently until fractures occur, osteoporosis significantly contributes to morbidity, mortality, long-term disability, and increased demands on healthcare systems, establishing it as a major global public health concern [4]. Recent assessments of the global burden of disease indicate a steady rise in mortality and disability attributable to low bone mineral density (BMD), including osteoporosis and related fractures, particularly among post-menopausal women [5]. In addition to its clinical ramifications, osteoporosis imposes a considerable economic burden worldwide, with direct medical costs, surgical interventions, rehabilitation, and productivity losses exerting significant strain on healthcare systems and societies [6–8].

Current therapeutic strategies for managing osteoporosis primarily fall into two categories: anti-resorptive and anabolic therapies. Anti-resorptive agents, including bisphosphonates, denosumab, and selective estrogen receptor modulators, inhibit osteoclast-mediated bone resorption, while anabolic treatments, such as romosozumab and teriparatide, focus on stimulating osteoblastic bone formation [9]. Despite their clinical efficacy, these medications are often limited by adverse reactions [10], prompting an ongoing search for more effective and safer pharmacological alternatives.

Unlike postmenopausal osteoporosis, which is primarily driven by estrogen deficiency, age-related (or senile) osteoporosis is characterized by a gradual decline in bone formation and quality. This condition involves the progressive accumulation of oxidative stress and senescence of mesenchymal stem cells in bone marrow, leading to a diminished pool of functional osteoblasts [11]. Key pathological features include reduced cortical thickness and increased marrow adiposity, often occurring in both sexes due to intrinsic cellular aging rather than hormonal withdrawal alone [12].

The primary objective of modeling age-related osteoporosis is to replicate the gradual decline in bone quality and progressive bone loss characteristic of elderly humans, thereby facilitating controlled investigations into molecular mechanisms, genetic influences, and potential therapeutic strategies [13, 14]. Rodent models, particularly mice and rats, serve as the cornerstone of preclinical research on osteoporosis. Their high genetic similarity to humans—over 75% of aging-related genes are conserved—combined with their manageable size, relatively short lifespan, and cost-effectiveness, renders them especially suitable for mechanistic and high-throughput studies [15]. Several aging mouse strains, such as naturally aged C57BL/6 and BALB/c, as well as accelerated senescence models like senescence-accelerated mouse prone 6 (SAMP6), exhibit spontaneous bone loss, trabecular thinning, cortical porosity, and other osteoporotic features, thereby recapitulating human skeletal aging [16]. In aged rats, declines in bone density, turnover, and microstructural integrity have also been documented, providing another physiologically relevant model [17]. Alternative models, including rabbits, tree shrews, pigs, sheep, and even teleost fish, are being explored; however, data on age-related osteoporosis in these species remain limited, and each model presents distinct practical or biological constraints [14, 18].

The D-galactose-induced osteoporosis model has emerged as a widely used accelerated aging paradigm for simulating senile osteoporosis in rodents. Chronic administration of D-galactose, a metabolic monosaccharide, disrupts redox homeostasis, elevates oxidative stress, impairs antioxidant defenses, and accelerates cellular senescence through markers such as tumor protein p53 (p53) and cyclin-dependent kinase inhibitor 1A (p21), thereby inducing skeletal alterations that closely resemble aging [19, 20]. Experimental evidence indicates that D-galactose treatment increases malondialdehyde (MDA) and advanced glycation end-product accumulation, enhances osteoclastogenesis, and impairs trabecular microarchitecture, with even greater effects observed when combined with metabolic stress, such as obesity [21, 22]. The primary advantages of this model are its relatively rapid induction of aging-like skeletal deterioration and its systemic relevance for preclinical research. However, considerable variability in dosing regimens (e.g., 150 mg/kg/day vs. 500 mg/kg/day) and treatment durations (e.g., 4 weeks vs. 8 weeks) across studies leads to inconsistent bone outcomes, hindering cross-study comparability and reproducibility [23, 24]. High doses may provoke systemic oxidative injury, particularly in the liver, with limited histopathological evidence and potential confounding effects [25]. Moreover, the progressive accumulation of advanced glycation end-products complicates the identification of optimal assessment windows, further limiting standardization. Although the D-galactose model remains valuable for investigating aging-related osteoporosis, harmonized protocols for dosing, duration, and outcome assessment are necessary to enhance reproducibility and translational relevance [26].

This scoping review aims to map and synthesize the current evidence on D-galactose-induced bone loss in rodents, with particular emphasis on study design, dosing strategies, treatment duration, and skeletal outcomes. By systematically identifying consistencies and discrepancies across the literature, this review seeks to highlight methodological gaps, propose recommendations for experimental design and reporting, and provide a clearer framework for the use of D-galactose in modeling senile osteoporosis. Ultimately, this review offers a comprehensive overview of the model’s strengths, limitations, and potential applications in osteoporosis research.

## Materials and methods

### Study framework and protocol registration

This scoping review was conducted following the methodological framework established by Arksey and O’Malley [27] and adhered to the Preferred Reporting Items for Systematic Reviews and Meta-Analyses Extension for Scoping Reviews (PRISMA-ScR) guidelines [28]. The investigation comprised five distinct phases: (1) identifying the research question; (2) identifying relevant studies; (3) study selection; (4) charting the data; and (5) collating, summarising, and reporting the findings. The study protocol is registered and accessible on the Open Science Framework (https://osf.io/y6g2x/overview).

**Figure 1.**
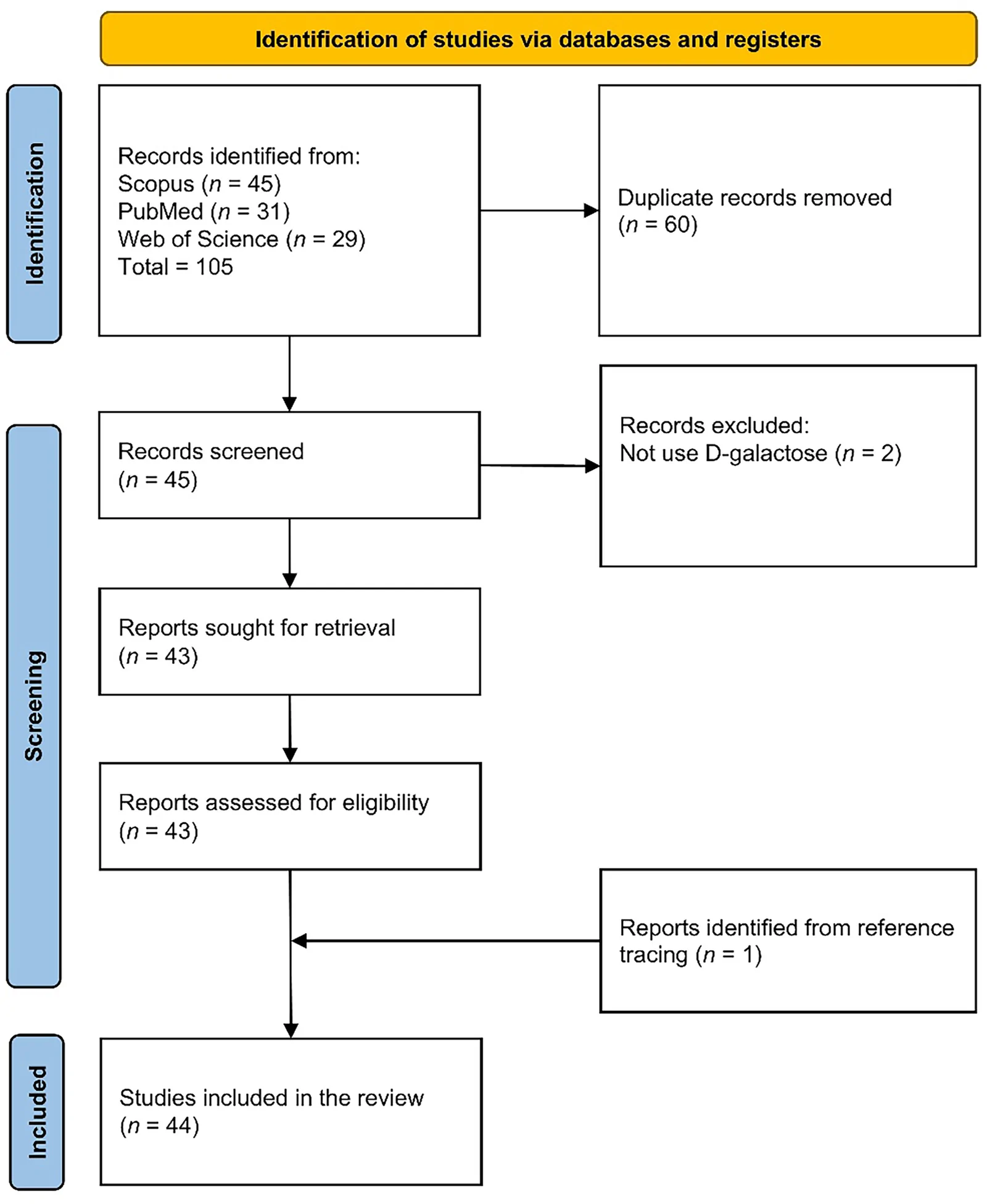
Flowchart illustrating the selection process for articles.

### Research question formulation

The review addressed the central question: “What is the optimal dose of D-galactose for inducing bone loss, and what are its specific effects on skeletal indices?” This inquiry was structured according to the Population, Intervention, Comparator, and Outcomes (PICO) framework (Table 1). Within this context, “optimal dose” refers to the specific quantity of D-galactose required to produce at least one statistically significant alteration in skeletal parameters, including densitometric, structural, mechanical, or mineral properties, in the experimental animals during the study period.

**Table 1:** Population, concepts, and context of the current scoping review

**Eligibility criteria**	**Description**
Population	Rodents Bone cells (osteoblasts, osteoclasts and their precursors)
Intervention	D-galactose
Comparator	Vehicle of D-galactose/Untreated
Outcomes	Bone health indices

### Literature search strategy

In August 2025, a comprehensive systematic search was performed across the PubMed, Scopus, and Web of Science databases. The search utilized a Boolean string: (Osteoporosis OR osteopenia OR “bone loss”) AND (D-galactose OR D-gal) AND (rats OR rat OR mouse OR mice). To ensure precision and avoid non-specific results, the search was restricted to titles and abstracts. No temporal filters were applied, thus encompassing all records from database inception to the date of the search.

Primary studies published in English or Mandarin that investigated the effects of D-galactose on bone loss were eligible for inclusion. Exclusion criteria applied to items lacking primary data, such as editorials, letters, reviews (including systematic reviews and meta-analyses), and perspectives. Conference abstracts and proceedings were also excluded due to incomplete data and potential duplication in full-length articles.

### Study selection and screening

Retrieved citations were imported into EndNote (version 21.4, Clarivate, London, UK) for automated deduplication. Following manual verification of the deduplication process, titles and abstracts were independently screened by L.L.G. and W.Y.Z. based on the predefined inclusion and exclusion criteria. Subsequently, full texts of eligible items were obtained and scrutinised by W.Y.Z. and K.-Y.C. To ensure literature saturation, the reference lists of included articles and relevant reviews were hand-searched to identify any additional eligible studies.

### Data charting process

Two researchers (K.-Y.C. and L.L.G.) extracted data using a standardized Google Form (Google, Mountain View, CA, USA). The extracted information included:

- Publication metadata: Authors and year of publication.
- Preclinical models: Species, strain, age, and sex.
- Experimental details: D-galactose dosage and induction period.
- Findings: Major outcomes related to bone health, limitations, and conclusions.

### Synthesis and reporting of results

Two independent reviewers (W.Y.Z. and L.L.G.) extracted study details for synthesis. The evidence was summarized and reported qualitatively, categorised by pathological indicators of bone loss. These included changes in bone densitometry, microstructure, cellularity, dynamics, mineral composition, and mechanical properties, alongside alterations in biochemical remodeling markers and molecular signaling pathways.

## Results

The literature search identified 45 unique articles from the three scholarly databases, of which 2 were excluded for not using D-galactose. One additional article was identified by examining the references of a previously selected study. In total, 44 articles were included in the current scoping review. The results of the article screening and selection are summarized in Figure 1.

### Study characteristics

A total of 42 studies employed *in vivo* models [19–24, 29–64], while two utilized *in vitro* models to investigate the effects of D-galactose on bone loss [65, 66]. Among the *in vivo* studies, 14 used Wistar rats aged 6–12 weeks [20–23, 31, 32, 36, 39, 47–49, 52, 60], although two studies did not specify the animals’ age [39, 48]. Eight studies involved Sprague-Dawley (SD) rats [29, 34, 44–46, 51, 63, 64], with ages ranging from five weeks to four months. Nine studies employed C57BL/6J mice aged 6–18 months [19, 24, 35, 37, 40, 41, 43, 54, 55]. Five studies used Institute of Cancer Research (ICR) mice [38, 58, 59, 61, 62], but three of which did not specify age [38, 58, 59]. The remaining studies reported ages of eight weeks [62] and three months [61]. Five studies utilized Kunming mice aged 8–17 weeks [42, 50, 53, 56, 57]. Seven studies employed combined models, including sex comparisons in SD rats [45], ovariectomy (OVX) combined with D-galactose in SD and Wistar rats [31, 46], D-galactose with sodium nitrite (NaNO_2_) in ICR mice [38], D-galactose with aluminium chloride in SD rats [34], D-galactose with periodontitis in C57BL/6J mice [43], and D-galactose with insulin in RAW 264.7 cells [21]. The remaining two *in vivo* studies did not specify either the strain/species or age of the rodents [30, 33]. For the *in vitro* studies, bone marrow mesenchymal stem cells (mBMSCs) were obtained from male C57BL/6J mice [66], and SD rats [65], both of unspecified age.

In the *in vivo* studies, D-galactose dosages ranged from 10–10,000 mg/kg/day, with 200 mg/kg/day being the most frequently used [19, 29, 30, 33, 35, 40, 47, 48]. Induction periods varied from 20 days to 5 months, with eight weeks being the most common [19, 23, 31–33, 35, 39, 40, 45, 47, 48, 55, 58, 59]. Regarding routes of administration, 18 studies employed intraperitoneal (i.p.) injections [23, 24, 29–33, 38–41, 44, 47, 48, 52, 58, 59, 62] 21 used subcutaneous (s.c.) injections [19, 20, 22, 34, 35, 37, 42, 43, 45, 46, 49–51, 53, 54, 56, 57, 60, 61, 63, 64], and two adopted oral administration [36, 55]. The fundamental characteristics of the included studies, covering animal models and D-galactose administration protocols, are summarized in Table 2.

**Table 2:** Summary of parameters for the D-galactose-induced aging model

**Category**	**Animal model**	**Dose and route**	**Induction period**	**Studies**
Standard rat model	Wistar/SD/undisclosed-strain rats, male, 3 months, 130–280 g	200 mg/kg/day, i.p.	8 weeks / 2 months	[29, 30, 33, 47, 48]
	Wistar rats, male/female, 6-12 weeks, 180–300 g	150 mg/kg/day, i.p. or s.c.	8–10 weeks	[20–23, 31]
	Wistar rats, male, 12 weeks (unspecified weight)	Diet containing 50% (w/w) galactose, p.o.	6.5 months	[36]
	SD rats, male, 2 months, 200 ± 10 g	125 mg/kg/day, s.c.	6 weeks	[51]
	Wistar rats, male, 12 weeks, 200 ± 20 g	120 mg/kg/day, i.p.	8 weeks	[32]
	SD rats, male and female, 3 months, 250–350 g	50–200 mg/kg/day, s.c.	2 months	[45]
	Wistar rats, male 230 ± 20 g	0.5 mL/kg (exact concentration not specified), i.p.	8 weeks	[39]
	Wistar rats, male, 8 weeks, 220 ± 20 g	750 mg/kg/day, i.p., 5 days/week	90 days	[52]
Standard mouse model	C57BL/6 mice, male, 6–8 weeks (unspecified weight)	200 mg/kg/day, s.c.	8 weeks	[19, 35]
	C57BL/6 mice, male, 18 months (unspecified weight)	200 mg/kg, i.p.	8 weeks	[40]
	C57BL/6 mice, male, 6 months (unspecified weight)	250 mg/kg/day, p.o.	8 weeks	[55]
	C57BL/6 mice, male, 6–8 weeks (unspecified weight)	150 mg/kg/day, s.c.	12 weeks	[54]
	C57BL/6J SPF mice, male, 8 weeks (unspecified weight)	500 mg/kg/day, i.p.	13 weeks	[24]
	C57BL/6 J SPF mice, male, 8 weeks, 18–20 g	500 mg/kg/day, s.c.	10 weeks	[43]
High-dose models	Kunming mice, female, 3-months, 30 ± 5 g)	1000 mg/kg/day, s.c.	42 days	[50]
	C57BL/6J mice, male, two and three months old, 30 ± 5 g	1000 mg/kg/day, i.p.	6 weeks	[41]
	Kunming mice, female, 13 weeks, 42 ± 5 g	10000 mg/kg/day, s.c.	20 days	[57]
	Wistar rats, male, 6 weeks old (unspecified weight)	1500 mg/kg/day, s.c.	5 months	[49]
Kunming/ICR models	ICR mice, male, 8–12 weeks, 20–45 g	100–150 mg/kg/day, i.p./s.c.	8–12 weeks	[38, 58, 59, 61, 62]
	Kunming mice, female/male, 8–17 weeks, 35–40 g	10–10000 mg/kg/day, s.c.	20–84 days	[42, 50, 53, 56, 57]
Long-term	SD rats, male, 4 months (unspecified weight)	125 mg/kg/day, i.p.	4 months	[44]
	SD rats, male, 9–12 weeks (unspecified weight)	100–125 mg/kg/day, s.c.	14–20 weeks	[63, 64]
OVX and combined	SD rats, OVX females, 3 months, 231.11 ± 18. 96 g	100 mg/kg/day, s.c.	60 days	[46]
*In vitro*	mBMSCs	0.03–100 mg/ml in culture	12 h–4 days	[65, 66]
	MC3T3-E1	100–400 mg/ml	24–48 h	[40]

**Abbreviations:** I.p.: intraperitoneal; ICR: Institute of Cancer Research; mBMSCs: mouse bone marrow mesenchymal stem cells; MC3T3-E1: mouse calvaria-derived preosteoblastic cell line; OVX: ovariectomized: P.o.: per os (oral administration); s.c.: subcutaneous; SD: Sprague-Dawley; SPF: specific pathogen-free; w/w: weight/weight.

### Rat models

D-galactose has been utilized to induce aging phenotypes and osteoporosis in various rat models through both dietary and injectable methods. In an early study, male Wistar rats were subjected to a 50% galactose diet for 6.5 months. This intervention impaired weight gain, suppressed serum osteocalcin (OCN) levels, increased urinary excretion of pyridinoline and deoxypyridinoline, and reduced bone volume and osteoblast numbers, implicating the polyol pathway in the underlying mechanisms [36].

Subsequent investigations primarily employed s.c. or i.p. injections. Long-term, high-dose s.c. administration (1500 mg/kg/day for 5 months) in young Wistar rats was reported to reduce BMD, compromise biomechanical properties, and alter bone mineral content [49]. Lower doses were also effective; for instance, s.c. injection of 125 mg/kg/day for 6–9 weeks in SD rats induced metabolic alterations and trabecular bone loss that resembled age-related osteoporosis [51]. Similarly, i.p. injection of 150 mg/kg/day for 8 weeks in Wistar rats resulted in trabecular deterioration and increased cortical porosity [23].

**Table 3:** Summary of studies on D-galactose-induced osteoporosis in rat models

**Authors (year)**	**Animal model**	**Treatment**	**Dosage/Duration**	**Key effects on bone**
Inaba et al. (1999) [36]	Male Wistar rats (12 weeks)	Galactose feeding	50% (wt/wt) galactose in MF diet, 6.5 months	Bone: ↓ BV/TV; ↓ N.Ob/BV Serum: ↓ OCN Urine: ↑ PYR, DPYR, Ca
Pei et al. [49]	Male Wistar rats (6 weeks)	D-gal (s.c.)	1500 mg/kg/day, 5 months	Bone: ↓ BMD, strength, minerals (Ca/Mn/Mg), hydroxyproline; ↑ ALP Serum: ↑ MDA, ↓ SOD, ↑ ALP, ↓ P
Ruan et al. (2009) [51]	Male SD rats (2 months)	D-gal (s.c.)	125 mg/kg/day, 6–9 weeks	Bone: ↓ trabeculae, bone formation Serum: ↓ PICP, ↓ Ca/Mg, ↑ OCN, ↑ P Urine: ↓ Ca/P, ↑ Mg
Imerb et al. (2022) [22]	Male Wistar rats (6 weeks)	D-gal (s.c.)	150 mg/kg/day, 8 weeks	Serum: ↑ AGEs, TNF-α, MDA; ↑ CTX-I, TRACP-5b; ↓ P1NP; ↑ glucose, insulin, HOMA-IR; ↑ TC, TG; ↓ HDL Bone: ↑ MDA; ↓ BV/TV, Tb.Th, Tb.N; ↑ Tb.Sp
Hung et al. (2014) [23]	Male Wistar rats (12 weeks)	D-gal (i.p.)	150 mg/kg/day, 8 weeks	Bone: ↓ Frame volume, ↓ Femur volume, ↑ Porosity, ↑ Frame density; no effect on stiffness/ultimate force
Zhang et al. [64]	Male SD rats (3 months)	D-gal (s.c.)	100 mg/kg/day, 14 weeks	Bone: ↓ BMD, Tb.Ar, Tb.Th; ↑ Tb.Sp, OCN; ↓ femoral Ca; ↓ elastic load
El-Baz et al. [33]	Male albino rats (unspecified age)	D-gal (i.p.)	200 mg/kg/day, 8 weeks	Bone: ↓ BMD, BV/TV, Tb.Th, Tb.N; ↑ osteoclasts Serum: ↑ Ca, BALP, IL-6, OPG/RANKL; ↓ P/Catalase
Li et al. [39]	Male Wistar rats (unspecified age)	D-gal (i.p.)	0.5 mL/kg/day, 8 weeks (exact concentration not specified)	Serum: ↑ ALOX15B and ↓ OCN
Xu et al. (2020) [60]	Male Wistar rats (8 weeks, 200±20 g)	D-gal (s.c.)	100 mg/kg/day, 4 months	Bone: ↓ BMD, BV/TV, Tb.N; ↑ Tb.Sp, BS/BV; ↓ FoxO1, SOD2 Serum: ↓ OCN, ALP, CAT, SOD, GSH-Px; ↑ MDA
Yu et al. (2020) [63]	Male SD rats (9 weeks, 200–250 g)	D-gal (s.c.)	100 mg/kg/day, 20 weeks	Bone: ↓ Tb.N, BV/TV, BMD; ↑ Tb.Sp; ↓ MAR, ↑ Oc.N; ↓ bone strength Serum: ↑ TRACP, MDA
Mahmoud et al. (2021) [47]	Male Wistar albino rats (3 months)	D-gal (i.p.)	200 mg/kg/day, 8 weeks	Bone: ↓ BMD, ERK expression Serum: ↑ Ca, ↓ P; ↓ SOD, CAT; ↑ BALP/OCN; ↓ OPG; ↑ RANKL, CTSK, TRAP
Imerb et al. (2022) [21]	Male Wistar rats (6 weeks)	D-gal (s.c.)	150 mg/kg/day, 10 weeks	Serum: ↑ MDA, AGEs, sRAGE; ↑ CTX-I Bone: ↓ BV/TV, Tb.N, Tb.Th; ↑ Tb.Sp, RANKL, CTX-I; ↑ TNF-α/IL-1β/IL-6
Mahmoud et al. (2022) [48]	Male Wistar albino rats (150–180 g)	D-gal (i.p.)	200 mg/kg/day, 8 weeks	Bone: ↓ BMD, AT2R, ↑ ERK, AT1R. Serum: ↑ Ca, ↓ P; ↑ BALP, OCN, ↓ OPG; ↑ RANKL, TRAP, CTSK; ↓ SOD, ↓ CAT
Dong et al. (2023) [32]	Male Wistar rats (12 weeks)	D-gal (i.p.)	120 mg/kg/day, 8 weeks	Bone: ↓ BMD, BVF, Tb.Th, Tb.N; ↑ Tb.Sp, BS/BV Serum: ↑ Hyp, ALP, MDA; ↓ TAOC, SOD
Imerb et al. (2023) [20]	Male Wistar rats (6 weeks)	D-gal (s.c.)	150 mg/kg/day, 10 weeks	Bone: ↑ p16/p21, HIF-1α/HIF-2α, TNF-α/IL-6; RANKL/RANK/NFATc1/DC-STAMP/CTSK. ↓ Osx, ALP, Col1a1, OPG/RANKL; ↓ BV/TV, Tb.Th, Ca/Mg Serum: ↑ insulin, ↑ HOMA-IR
Shen et al. (2024) [52]	Male Wistar rats (8 weeks)	D-gal (i.p.)	750 mg/kg/day, 5 day/week, 90 days	Bone: ↓ BV/TV, vBMD Serum: ↑ FlOP_320, ↑ MDA; ↓ P1NP
Baseem et al. (2024) [30]	Male rats (unspecified age)	D-gal (i.p.)	200 mg/kg/day, 60 days	Bone: ↓ trabecular thickness, ↑ trabecular resorption, thinner trabeculae, ↑ osteoclasts Serum: ↑ RANK, RANKL, cathepsin K, ↓ OPG
Ali and Abdulazeez, 2024 [29]	Male SD rats (3 months, 280±10 g)	D-gal (i.p.)	200 mg/kg/day, 60 days	Bone: ↓ trabecular thickness, ↑ microfractures Serum: ↑ ALP, OC

**Abbreviations:** ↑: increase; ↓: decrease; AGEs: advanced glycation end products; ALOX15B: arachidonate 15-lipoxygenase type B; ALP: alkaline phosphatase; AT1R: angiotensin II type 1 receptor; AT2R: angiotensin II type 2 receptor; BALP: bone-specific alkaline phosphatase; BMD: bone mineral density; BS/BV: bone surface/bone volume; BV/TV: bone volume/tissue volume; BVF: bone volume fraction; Ca: calcium; CAT: catalase; Col1a1: collagen type I alpha 1 chain; CTSK: cathepsin K; CTX-I: C-terminal telopeptide of type I collagen; D-gal: D-galactose; DC-STAMP: dendritic cell-specific transmembrane protein; DPYR: deoxypyridinoline; ERK: extracellular signal-regulated kinase; FlOP_320, fluorescent oxidation products at 320 nm; FoxO1, forkhead box O1; GSH-Px, glutathione peroxidase; HDL, high-density lipoprotein; HIF-1α: hypoxia-inducible factor 1 alpha; HIF-2α: hypoxia-inducible factor 2 alpha; HOMA-IR: homeostatic model assessment of insulin resistance; Hyp: hydroxyproline: I.p.: intraperitoneal; IL-1β: interleukin 1 beta; IL-6: interleukin 6; MDA: malondialdehyde; Mg: magnesium; Mn: manganese: N.Ob/BV: number of osteoblasts per bone volume; NFATc1: nuclear factor of activated T cells 1; OCN: osteocalcin: Oc.N: osteoclast number; OPG: osteoprotegerin; Osx: osterix; P: phosphorus; P1NP: procollagen type I N-terminal propeptide; PICP: procollagen type I C-terminal propeptide; PYR: pyridinoline; RANK: receptor activator of nuclear factor κB; RANKL: receptor activator of nuclear factor κB ligand: S.c.: subcutaneous; SD: Sprague-Dawley; SOD: superoxide dismutase; SOD2: superoxide dismutase 2; sRAGE: soluble receptor for advanced glycation end products; TAOC: total antioxidant capacity: Tb.Ar: trabecular area: Tb.N: trabecular number: Tb.Sp: trabecular separation: Tb.Th: trabecular thickness; TC: total cholesterol; TG: triglycerides; TNF-α: tumor necrosis factor alpha; TRACP: tartrate-resistant acid phosphatase; TRACP-5b: tartrate-resistant acid phosphatase 5b; vBMD: volumetric bone mineral density; wt/wt: weight/weight.

The osteoporotic phenotype was consistently characterized by microarchitectural deterioration. Following s.c. administration of 100 mg/kg/day for 14 weeks in SD rats, researchers observed reduced BMD, trabecular bone volume (BV/TV), and trabecular thickness (Tb.Th), along with increased trabecular separation (Tb.Sp) and osteoclast numbers [64].

Altered bone turnover was frequently reported, evidenced by decreased formation markers (e.g., OCN, alkaline phosphatase (ALP), procollagen type I N-terminal propeptide (P1NP)) and increased resorption markers (e.g., C-terminal telopeptide of type I collagen (CTX-I), tartrate-resistant acid phosphatase (TRAP), receptor activator of nuclear factor-κB ligand (RANKL)) following i.p. treatment at 200 mg/kg/day for 8 weeks [33, 47, 48]. Prolonged treatment (100 mg/kg/day s.c. for 20 weeks) resulted in oxidative stress, indicated by increased MDA, decreased superoxide dismutase (SOD), decreased catalase (CAT), elevated serum TRAP, and impaired bone strength [63].

The role of oxidative stress was emphasized across numerous studies, with consistent evidence of lipid peroxidation (increased MDA) and reduced antioxidant enzyme activity (SOD, CAT) in D-galactose-treated rats [32, 47, 48, 52]. High-dose intermittent administration (750 mg/kg/day i.p., 5 days/week for 90 days) corroborated these findings, additionally revealing suppressed bone formation through reduced P1NP [52].

Beyond structural and biochemical alterations, cellular mechanisms were implicated. Increased expression of senescence markers, specifically cyclin-dependent kinase inhibitor 2A (p16) and p21, along with hypoxia-inducible factors 1 alpha (HIF-1α) and 2 alpha (HIF-2α), and inflammatory mediators, was observed in Wistar rats following D-galactose treatment (150 mg/kg/day for 10 weeks, s.c.). This was accompanied by a reduction in osteoblast gene expression and deterioration of trabecular microarchitecture [20]. This model was also validated for pharmacological applications, including pharmacokinetic investigations of anti-osteoporotic formulations [39]. Several agents, including diosgenin [23], telmisartan [48], cycloastragenol [63], and herbal extracts [32], were found to mitigate D-galactose-induced bone loss. Recent studies further confirmed the model’s robustness; at a dose of 200 mg/kg/day (i.p.) for 60 days, the model induced osteoporosis-like changes characterized by enhanced RANKL/receptor activator of nuclear factor-κB (RANK) signaling, elevated turnover markers, trabecular thinning, and cortical resorption [29, 30]. The characteristics and applications of D-galactose-induced osteoporotic models in rats are summarized in Table 3.

**Table 4:** Overview of studies on D-galactose-induced osteoporosis in mouse models

**Authors (year)**	**Animal model**	**Treatment and duration**	**Key effects on bone**
Qin et al. (2003) [50]	Female Kunming mice, 3 months	D-gal, 1000 mg/kg/day, 42 days (s.c.)	Bone: ↓ Bone weight, Hyp, Ca, Mg Serum: ↓ GSH-Px, SOD, CAT; ↑ MDA
Situ et al. (2014) [53]	Female Kunming mice, 2 months	D-gal, 120 mg/kg/day, 70 days (s.c.)	Bone: ↓ Tb.Ar, Tb.Th, Tb.N; ↑ Tb.Sp, osteoclast activity; ↓ bone formation
Liang et al. (2019) [42]	Male Kunming mice (35–40g), 17 weeks	D-gal, 150 mg/kg/day, 12 weeks (s.c.)	Bone: ↓ BMD, BV/TV, Tb.N, Conn.D; ↑ SMI
Liu et al., 2024 [43]	Male C57BL/6J mice (8 weeks)	D-gal, 500 mg/kg/day, 10 weeks (s.c.)	Alveolar bone: ↑ ABL, ↓ alveolar ridge height; ↑ TRAP^+^ osteoclasts; inflammatory infiltration Serum: ↑ TNF-α, IL-6; ↓ SOD, ↑ MDA Gut barrier: ↓ occludin, ZO-1 (barrier disruption) Microbiota/metabolites: ↓ F/B ratio; dysbiosis (↑ Bacteroides, etc.); altered lipid and amino acid metabolism (e.g., histidine pathway)
Wu et al. (2021) [56]	Female Kunming mice, 10 weeks	D-gal 10 mg/kg/day + low-Ca diet, 20 days (s.c.)	Bone: ↓ BMD, Tb.N, connectivity; bone dry/wet ratio
Wu et al. (2021) [57]	Female Kunming mice, 13 weeks	D-gal 10000 mg/kg/day + low-Ca diet, 20 days (s.c.)	Bone: ↓ BMD, BV/TV, Tb.N, Tb.Th; ↑ Tb.Sp, BS/BV; ↓ bone index and dry/wet ratio Oxidative status: ↓ SOD, ↑ MDA (liver/kidney) Calcium metabolism: ↑ urinary Ca/Cr, urinary Ca; ↓ fecal Ca
Hsu et al. (2020) [35]	Male C57BL/6 mice, 6 weeks	D-gal, 200 mg/kg/day, 8 weeks (s.c.)	Brain: ↑ ROS, MDA; ↓ SOD, CAT, GSH-Px; ↑ p16, p53, p21; ↓ SIRT1; ↓ neurogenesis (↓ BrdU, DCX, NeuN); ↑ GFAP, Iba1, TNF-α; ↓ PI3K/Akt signaling Serum: ↑ Glu, BUN, LDH, AST, ALT, TG, TC; MDA; ↓ SOD, CAT, GSH-Px
Su et al., 2022 [54]	Male C57BL/6 mice, 6–8 weeks	D-gal, 150 mg/kg/day, 12 weeks (s.c.)	Bone: ↓ BMD, BV/TV, Tb.N; ↑ Tb.Sp, SMI; ↓ stiffness and max load; ↓ LC3II/Beclin-1, ↑ p62; ↓ Ki-67, ↑ p16; ↑ Bax and Cleaved Caspase-3, ↓ Bcl-2 Serum: ↑ IL-1β, IL-6, MDA
Wang et al., 2022 [55]	Male C57BL/6J mice, 6 months	Oral D-gal, 250 mg/kg/day, 8 weeks	Bone: ↓ BMD, BV/TV, Tb.N; ↑ Tb.Sp; Serum: ↑ MDA, ↓ SOD, ALP, OCN, OPN
Wang et al., 2022 [19]	Male C57BL/6J mice, 8 weeks	D-gal, 200 mg/kg/day, 8 weeks (s.c.)	Bone: ↓ BMD; ↑ RANKL, RANK, c-Fos; ↑ NF-κB activation (↑ p-IκBα, nuclear p65); ↓ OPG; ↑ p53 Serum: ↑ OCN
Xu et al., 2022 [61]	Male ICR mice, 8 weeks	D-gal, 150 mg/kg/day, 12 weeks (s.c.)	Bone: ↓ BMD, BV/TV, Tb.N, Tb.Th; MAR, BFR/BS; ↑ P16/P21/γ-H2A.X; ↑ SASP (MMP3, MMP13, IL-1α, IL-6, TNF-α); ↑ lipid peroxidation (↑ MDA, ↑ 4-HNE) Serum: ↓ 1,25(OH)_2_D_3_, GSH
Xu et al., 2022 [62]	Male ICR mice, 3 months	D-gal, 150 mg/kg/day, 12 weeks (i.p.)	Bone: ↓ BMD, BV/TV, Tb.N, Conn.D; ↑ Tb.Sp, SMI Serum: ↓ ALP, OCN; ↑ TRACP; ↓ SOD, CAT
Xiong et al., 2022 [58]	Male ICR mice (unspecified age)	D-gal, 120 mg/kg/day, 8 weeks (i.p.)	Bone: ↓ trabecular number/connectivity, ↓ bone area and mineralisation; trabecular thinning/fracture (microstructural deterioration) Serum: ↓ Ca, P, CAT; ↑ ALP
Hu et al., 2024 [24]	Male C57BL/6J mice, 8 weeks	D-gal, 500 mg/kg/day, 13 weeks (i.p.)	Systemic: ↓ body weight, ↓ spleen/thymus index Alveolar bone: ↑ bone loss, ↑ osteoclasts (↑ TRAP), ↓ alveolar crest height Serum: ↓ SOD, ↑ MDA, ↑ IL-6, ↑ TNF-α Intestine/Gut: ↓ ZO-1, ↓ occludin (barrier impairment); ↓ α-diversity, altered microbiota (↓ F/B ratio)
Kaushal et al., 2024 [37]	Male C57BL/6J mice, 20 weeks	D-gal, 100 mg/kg/day, 10 weeks (s.c.)	Bone: ↓ BV/TV, Tb.N, Tb.Th, ↑ Tb.Sp; ↓ bone strength; ↓ P1NP, ↑ CTX; ↓ Runx-2/Col-1; ↑ p53/p21/pRB (senescence) Immune/Serum: ↑ PTH, ↑ β-gal; ↑ Th17 (↑ IL-17, ↑ ROR-γt, ↑ STAT-3), ↓ Treg (↓ Foxp3); ↑ TNF-α
Li et al., 2024 [40]	Male C57BL/6J mice, 18 months	D-gal, 200 mg/kg/day, 8 weeks (i.p.)	Bone: ↓ BMD; Tb.BV/TV, Tb.N, Tb.Th, ↑ Tb.Sp; ↓ Ct.Th, Conn.D; ↓ collagen deposition; ↓ TRIM16, Runx-2/Col-1/OPN
Li et al. (2024) [41]	Male C57BL/6J mice, 2–3 months	D-gal, 1000 mg/kg/day, 6 weeks (i.p.)	Bone: ↓ BV/TV, BMD, Tb.N, Ct.Th; ↑ Tb.Sp; ↑ senescence (p16, p53)
Xiong et al. (2024) [59]	Male ICR mice (unspecified age)	D-gal, 100 mg/kg/day, 8 weeks (i.p.)	Bone: ↓ femur length, ↓ femur and tibia dry weight Serum: ↓ Ca, ↓ Pi; ↑ ALP; ↓ CAT activity

**Abbreviations:** ↑: increase; ↓: decrease; 1,25(OH)_2_D_3_: 1,25-dihydroxyvitamin D_3_; 4-HNE: 4-hydroxynonenal; ABL: alveolar bone loss; ALP: alkaline phosphatase; ALT: alanine aminotransferase; AST: aspartate aminotransferase; Bax: Bcl-2-associated X protein; Bcl-2: B-cell lymphoma 2; BFR/BS: bone formation rate/bone surface; BMD: bone mineral density; BrdU: bromodeoxyuridine; BS/BV: bone surface/bone volume; BUN: blood urea nitrogen; BV/TV: bone volume/tissue volume; Ca: calcium; CAT: catalase; c-Fos: cellular Fos; Col-1: collagen type I: Conn.D: connectivity density: Ct.Th: cortical thickness; CTX: C-terminal telopeptide of type I collagen; D-gal: D-galactose; DCX: doublecortin; F/B ratio: Firmicutes/Bacteroidetes ratio; Foxp3: forkhead box P3; GFAP: glial fibrillary acidic protein; Glu: glucose; GSH: reduced glutathione; GSH-Px: glutathione peroxidase; Hyp: hydroxyproline; Iba1: ionized calcium-binding adaptor molecule 1; ICR: Institute of Cancer Research: I.p.: intraperitoneal; IL-1α: interleukin 1 alpha; IL-1β: interleukin 1 beta; IL-6: interleukin 6; IL-17: interleukin 17; Ki-67: marker of cell proliferation Ki-67; LC3II: microtubule-associated protein 1 light chain 3 II; LDH: lactate dehydrogenase; MAR: mineral apposition rate; MDA: malondialdehyde; Mg: magnesium; MMP3: matrix metalloproteinase 3; MMP13: matrix metalloproteinase 13; NF-κB: nuclear factor kappa B; NeuN: neuronal nuclei; OCN: osteocalcin; OPG: osteoprotegerin; OPN: osteopontin; p16: cyclin-dependent kinase inhibitor 2A; p21: cyclin-dependent kinase inhibitor 1A; p53: tumor protein p53; p62: sequestosome 1; p65: nuclear factor κB p65 subunit; p-IκBα: phosphorylated inhibitor of κB alpha; P1NP: procollagen type I N-terminal propeptide; Pi: inorganic phosphate; PI3K/Akt: phosphoinositide 3-kinase/protein kinase B; pRB: retinoblastoma protein; PTH: parathyroid hormone; RANK: receptor activator of nuclear factor κB; RANKL: receptor activator of nuclear factor κB ligand; ROR-γt: retinoic acid receptor-related orphan receptor gamma t; ROS: reactive oxygen species; Runx-2: runt-related transcription factor 2; SASP: senescence-associated secretory phenotype: S.c.: subcutaneous; SIRT1: sirtuin 1; SMI: structure model index; SOD: superoxide dismutase; STAT-3: signal transducer and activator of transcription 3: Tb.Ar: trabecular area: Tb.BV/TV: trabecular bone volume/tissue volume: Tb.N: trabecular number: Tb.Sp: trabecular separation: Tb.Th: trabecular thickness; TC: total cholesterol; TG: triglycerides; Th17: T helper 17 cells; TNF-α: tumor necrosis factor alpha; TRACP: tartrate-resistant acid phosphatase; TRAP: tartrate-resistant acid phosphatase; Treg: regulatory T cells; TRIM16: tripartite motif containing 16; ZO-1: zonula occludens 1; β-gal: beta-galactosidase; γ-H2A.X: phosphorylated histone H2A.X.

### Mouse models

The D-galactose-induced aging model has been extensively established in various mouse strains, demonstrating its utility for studying age-related osteoporosis. In female Kunming mice, s.c. injection of a high dose (1000 mg/kg/day for 42 days) resulted in significant bone loss associated with decreased bone magnesium content, impaired antioxidant defenses, and increased lipid peroxidation [50]. A longer protocol with a lower dose (120 mg/kg/day for 70 days) also led to deterioration of trabecular bone microstructure, enhanced osteoclastic activity, and reduced bone formation rates, mirroring the effects of OVX [53].

In male Kunming mice, s.c. D-galactose administration (150 mg/kg/day for 12 weeks) successfully induced an osteoporotic phenotype characterized by reduced BMD, BV/TV, trabecular number (Tb.N), and connectivity density (Conn.D), alongside an increased structural model index (SMI) [42]. A combined regimen of D-galactose with a low-calcium diet (10 mg/kg/day s.c. plus a 0.1% Ca diet for 20 days) in female Kunming mice further exacerbated bone loss, reflected in impaired trabecular microarchitecture and decreased trabecular number and density [56, 57].

In C57BL/6 and ICR mice, s.c. or i.p. injections of 150–200 mg/kg/day for 8–12 weeks consistently produced osteoporotic features, including reduced BMD, deteriorated trabecular microarchitecture, increased oxidative stress (decreased SOD, increased MDA), and elevated senescence and apoptosis markers [19, 54, 55, 58, 61, 62]. Bone deterioration was also associated with impaired osteoblast function and suppression of the nuclear factor erythroid 2-related factor 2 (Nrf2)/glutathione peroxidase 4 (GPX4) antioxidant pathway, contributing to oxidative stress and ferroptosis [61, 62]. This model has frequently been employed to evaluate therapeutic interventions, with agents such as glucosamine [54], alginate oligosaccharide [19], and bitter acids from *Humulus lupulus *L. [62] reported to mitigate bone loss.

Oral administration of D-galactose (250 mg/kg/day for 8 weeks) also induced osteoporosis in male C57BL/6J mice through oxidative stress, which was partially reversed by treatment with human umbilical cord cells [55]. Furthermore, i.p. administration of D-galactose (500 mg/kg/day for 13 weeks) was utilized to model alveolar bone loss, leading to oxidative stress, inflammation, immune dysfunction, and osteoclast-mediated alveolar resorption [24].

Recent studies have further validated and refined the model. D-galactose (100 mg/kg/day s.c. for 10 weeks) in 20-week-old male C57BL/6J mice induced osteoporosis associated with high bone turnover, senescence, oxidative stress, and a T-helper 17 (Th17)-driven pro-inflammatory response [37]. In older mice (18 months), i.p. D-galactose (200 mg/kg for 8 weeks) exacerbated age-related bone loss and suppressed osteogenic markers [40], while a higher dose (1000 mg/kg/day for 6 weeks) in young adult mice caused severe trabecular and cortical deterioration, mimicking senile osteoporosis [41]. Similarly, i.p. administration of 100 mg/kg/day for 8 weeks in ICR mice induced features similar to senile osteoporosis (SOP), including mineral imbalances, elevated ALP, and trabecular deterioration [59]. The characteristics and applications of D-galactose-induced osteoporotic models reported across these studies are summarized in Table 4.

**Table 5:** Summary of studies on D-galactose-induced osteoporosis utilizing combined models

**Authors (year)**	**Model type**	**Model details**	**Treatment**	**Dosage/duration**	**Key effects on bone**
Luo et al. (2004) [45]	Sex comparison	Male and female SD rats, 3 months old	D-gal (s.c.)	50, 100, 200 mg/kg/day, 2 months	Males: ↓ Tb.Ar, Tb.N, Tb.Th, ↑ Tb.Sp; ↓ %L.Pm, ↓ MAR, ↓ bone strength Females: no significant skeletal changes
Lyu et al. (2016) [46]	OVX + D-gal	Female SD rats, 3 months old (OVX)	D-gal (s.c.)	100 mg/kg/day, 60 days	Bone: ↓ BV/TV, Tb.N, Tb.Th, BMD, Conn.Dens; ↑ Tb.Sp, SMI; ↓ maximum load; ↓ stiffness, maximum strength, break strength, elastic modulus; ↓ Ca, P; ↓ Hyp
Li et al. (2019) [38]	D-gal + NaNO_2_	ICR mice	D-gal (i.p.) + NaNO_2_	120 mg/kg/day, 90 days	Bone: ↓ BV/TV, Tb.N, Tb.Th, Tt.Ar, Ct.Ar, Ct.Th, bone mineral/matrix ratio; ↑ Tb.Sp, ↑ CTx-1, ↓ P1NP Serum: ↑ MDA, 8-OH-dG, ↓ TAC, GSH/GSSG; ↑ TNF-α, ↓ IL-2; ↑ TMAO, FMO3
Chen et al. (2020) [31]	OVX + D-gal + AlCl_3_	Female Wistar rats, 8 weeks (OVX)	D-gal (i.p.) + AlCl_3_ (p.o.)	150 mg/kg/day D-gal + 30 mg/kg/day AlCl_3_, 8 weeks	Bone: ↓ BMD, BV/TV, Tb.N, Tb.Th, BS/TV; ↑ Tb.Sp, ↑ SMI, ↑ Tb.Pf; ↓ maximum load, maximum stress, energy absorption; ↑ BALP, TRAP Serum: ↓ SOD, ↑ MDA; ↑ BALP, ↑ TRAP Hippocampus: ↑ AChE, ↓ ChAT; ↓ SOD, ↑ MDA
He et al. (2022) [34]	D-gal + AlCl_3_	Male SD rats, 5 weeks	D-gal (s.c.) + AlCl_3_	D-gal 120 mg/kg/day + AlCl_3_ 10 mg/kg/day, 14 weeks	Alveolar bone: ↓ BV/TV; ↑ alveolar bone resorption height Serum / metabolism: ↑ TG, ↑ AST, ↑ ALT, ↑ AST/ALT; ↓ HDL-C Gut microbiota: ↑ Firmicutes/Bacteroidetes ratio; ↑ Aerococcus, ↑ Streptococcus; ↓ SCFA-producing bacteria diversity Brain (cortex and hippocampus): ↑ escape latency; ↓ platform crossings; ↑ Aβ1–42 deposition; ↓ neuronal density
Imerb et al. (2022) [22]	*In vitro*	RAW 264.7 cells	D-gal ± insulin	D-gal 5 mg/mL, insulin 100 nM	↑ TRAP^+^ OCs; ↑ osteoclastogenesis;
	*In vivo*	Male Wistar rats (6 weeks)	D-gal (s.c.)	150 mg/kg/day, 10 weeks	Serum: ↑ MDA/ AGEs/sRAGE; ↑ CTX-I Bone: ↓ BV/TV, Tb.N, Tb.Th, ↑ Tb.Sp, RANKL, CTX-I; TNF-α/IL-1β/IL-6
Li et al. (2020) [65]	*In vitro*	Rat BMSCs	D-gal	0.03mg/ml, 12h–14 days	↓ Cell proliferation; ↓ CYP27B1; ↑ CYP24A1; ↓ osteogenesis
	*In vivo*	Male SD rats(180±20g)	Astragali Radix	10 000mg /kg,2 times/day,7days	Preparation of Astragali Radix-containing serum
Liu et al. (2020) [44]	*In vivo*	Male SD rats; 4 months	D-gal (i.p.)	*In vivo*: 125 mg/kg/day, 4 months	↓ BV/TV, Tb.N, Tb.Th, ↑ Tb.Sp; ↓ maximum force, ultimate stress, Young’s modulus
	*In vitro*	rBMSCs (from D-galactose-treated rats)	/	/	↑ mTORC1/p70S6K/4EBP activation; ↑ H3K9me3, HP1γ, p53, PDT
Xu et al., 2022 [61]	*In vitro*	MC3T3-E1 and primary osteoblasts	D-gal	100 mM, 48 h	↑ mtROS, ↑ lipid peroxidation, ↑ PTGS2, ↑ P16/P21/γ-H2A.X, ↑ SASP genes (MMP3, MMP13, IL-1α, IL-6, TNF-α), ↑ SA-β-gal; ↓ viability, ↓ GSH/GSSG, ↓ ALP
	*In vivo*	Male ICR mice, 8 weeks	D-gal	150 mg/kg/day, 12 weeks (s.c.)	Bone: ↓ BMD, BV/TV, Tb.N, Tb.Th; MAR, BFR/BS; ↑ P16/P21/γ-H2A.X; ↑ SASP (MMP3, MMP13, IL-1α, IL-6, TNF-α); ↑ lipid peroxidation (↑ MDA, ↑ 4-HNE) Serum: ↓ 1,25(OH)_2_D_3_, GSH
Li et al., 2024 [40]	*In vitro*	MC3T3-E1 osteoblasts	D-gal	10–40 mg/ml, 24–48 h	↑ senescence (↑ p16/p21/p53, SA-β-gal), ↑ apoptosis; ↓ ALP, mineralisation; ↑ ROS; ↓ Nrf2/HO-1/SOD1; ↓ SOD, GSH; ↑ MDA
Liu et al., 2024 [43]	D-gal + periodontitis	Male C57BL/6J mice, 8 weeks	D-gal (s.c.) + periodontitis	500 mg/kg/day, 10 weeks	Alveolar bone: ↑ ABL, ↓ alveolar ridge height; ↑ TRAP^+^ osteoclasts; inflammatory infiltration Serum: ↑ TNF-α, IL-6; ↓ SOD, ↑ MDA Gut barrier: ↓ occludin, ZO-1 (barrier disruption) Microbiota/metabolites: ↓ F/B ratio; dysbiosis (↑ Bacteroides, etc.); altered lipid and amino acid metabolism (e.g., histidine pathway)
Liu et al. (2024) [66]	*In vitro*	Mouse BMSCs	D-gal	20 mg/ml, 24 h	↓ proliferation, ↓ osteogenic differentiation, ↑ apoptosis; ↑ iron and ferritin
	*In vivo*	hepcidin1-knockout aging mice	/	18 months (not treated with D-galactose)	Bone: ↓ Tb.N, BV/TV, BMD, ↑ Tb.Sp; ↓ OCN, ALP, Runx2, SP7, Col-1; ↑ RANKL, Mmp9, Trap, CTSK, OPG Serum: ↑ TRACP, ↑ ferritin, ↑ MDA, ↓ OCN, ↓ SOD,

**Abbreviations:** ↑: increase; ↓: decrease; 1,25(OH)_2_D_3_: 1,25-dihydroxyvitamin D_3_; 4EBP: eukaryotic translation initiation factor 4E-binding protein; 4-HNE: 4-hydroxynonenal; 8-OH-dG: 8-hydroxy-2′-deoxyguanosine; Aβ1–42: amyloid beta 1–42; ABL: alveolar bone loss; AChE: acetylcholinesterase; AGEs: advanced glycation end products; ALP: alkaline phosphatase; AlCl_3_: aluminium chloride; ALT: alanine aminotransferase; AST: aspartate aminotransferase; BALP: bone-specific alkaline phosphatase; BFR/BS: bone formation rate/bone surface; BMD: bone mineral density; BMSCs: bone marrow mesenchymal stem cells; BS/TV: bone surface/tissue volume; BV/TV: bone volume/tissue volume; Ca: calcium; CAT: catalase; ChAT: choline acetyltransferase; Col-1: collagen type I: Conn.Dens: connectivity density: Ct.Ar: cortical area: Ct.Th: cortical thickness; CTx-1/CTX-I: C-terminal telopeptide of type I collagen; CYP24A1: cytochrome P450 family 24 subfamily A member 1; CYP27B1: cytochrome P450 family 27 subfamily B member 1; D-gal: D-galactose; F/B ratio: Firmicutes/Bacteroidetes ratio; FMO3: flavin-containing monooxygenase 3; GSH: reduced glutathione; GSH/GSSG: reduced glutathione/oxidized glutathione ratio; H3K9me3: trimethylated histone H3 lysine 9; HDL-C: high-density lipoprotein cholesterol; HO-1: heme oxygenase 1; HP1γ: heterochromatin protein 1 gamma; Hyp: hydroxyproline; ICR: Institute of Cancer Research; IL-1α: interleukin 1 alpha; IL-1β: interleukin 1 beta; IL-2: interleukin 2; IL-6: interleukin 6: I.p.: intraperitoneal; MAR: mineral apposition rate; MC3T3-E1: mouse calvaria-derived preosteoblastic cell line; MDA: malondialdehyde; MMP3: matrix metalloproteinase 3; MMP13: matrix metalloproteinase 13; MMP9: matrix metalloproteinase 9; mtROS: mitochondrial reactive oxygen species; mTORC1: mechanistic target of rapamycin complex 1; NaNO_2_: sodium nitrite; Nrf2: nuclear factor erythroid 2-related factor 2; OCN: osteocalcin; OCs: osteoclasts; OPG: osteoprotegerin; OVX: ovariectomized: P.o.: per os (oral administration); p16: cyclin-dependent kinase inhibitor 2A; p21: cyclin-dependent kinase inhibitor 1A; p53: tumor protein p53; p70S6K: ribosomal protein S6 kinase beta-1; P1NP: procollagen type I N-terminal propeptide; PDT: population doubling time; PTGS2: prostaglandin-endoperoxide synthase 2; RANKL: receptor activator of nuclear factor κB ligand: RAW 264;7: murine macrophage cell line; ROS: reactive oxygen species; Runx-2/Runx2: runt-related transcription factor 2; SA-β-gal: senescence-associated beta-galactosidase; SASP: senescence-associated secretory phenotype; SCFA: short-chain fatty acid; SD: Sprague-Dawley: S.c.: subcutaneous; SOD: superoxide dismutase; SOD1: superoxide dismutase 1; SP7: Sp7 transcription factor SOD: superoxide dismutase; SOD1: superoxide dismutase 1; SP7: Sp7 transcription factor (osterix); Tb.Ar: trabecular area: Tb.N: trabecular number: Tb.Pf: trabecular pattern factor: Tb.Sp: trabecular separation: Tb.Th: trabecular thickness; TAC: total antioxidant capacity; TG: triglycerides; TMAO: trimethylamine N-oxide; TNF-α: tumor necrosis factor alpha; TRACP: tartrate-resistant acid phosphatase; TRAP: tartrate-resistant acid phosphatase: Tt.Ar: total area; ZO-1: zonula occludens 1.

### Combined models

To model complex forms of age-related and secondary osteoporosis, D-galactose was frequently combined with various interventions or applied in *in vitro* systems. One study highlighted a pronounced sexual dimorphism, wherein subcutaneously administered D-galactose (50–200 mg/kg/day for 2 months) induced trabecular bone loss and decreased bone formation rates in male SD rats, while significant changes were not observed in females of the same strain, suggesting a role of gonadal function [45].

To simulate postmenopausal or senile osteoporosis, D-galactose was administered in combination with OVX. In female SD rats, subcutaneous D-galactose (100 mg/kg/day) post-OVX exacerbated bone deterioration, as evidenced by diminished biomechanical strength, reduced mineral content (calcium, phosphate), hydroxyproline levels, and impaired trabecular microarchitecture [46]. Similarly, in female Kunming mice, D-galactose (120 mg/kg/day for 70 days) induced bone loss comparable to OVX, which was mitigated by estrogen treatment [53].

More complex multifactorial approaches have also been established. In female Wistar rats, a combination of OVX, intraperitoneal D-galactose (150 mg/kg/day), and oral aluminum chloride (AlCl3) over eight weeks resulted in significant bone loss, elevated turnover markers [bone-specific alkaline phosphatase (BALP), TRAP], oxidative stress, and hippocampal neuronal impairment, thereby providing a dual model of osteoporosis with Alzheimer-like pathology [31]. In ICR mice, intraperitoneal D-galactose combined with sodium nitrite for 90 days induced bone loss associated with gut dysbiosis, increased trimethylamine-N-oxide levels, and oxidative stress [38]. Other studies have utilized D-galactose alongside a high-fat diet, demonstrating that obesity-related aging exacerbates bone deterioration through oxidative stress, inflammation, and enhanced osteoclastogenesis [21, 22].

Specific contexts of bone loss have also been investigated. In male SD rats, combined treatment with subcutaneous D-galactose and AlCl3 resulted in alveolar bone loss and reduced BV/TV in an Alzheimer-like model [34]. In male C57BL/6J mice, D-galactose-induced aging aggravated ligature-induced periodontitis, leading to alveolar bone resorption, increased osteoclast activity, and alterations in gut microbiota and intestinal barrier function [39].

*In vitro* studies have further elucidated the underlying mechanisms. Rat BMSCs exposed to D-galactose (0.03 mg/ml) exhibited senescence, impaired osteogenic differentiation, and dysregulated vitamin D metabolism characterized by reduced cytochrome P450 family 27 subfamily B member 1 (CYP27B1) and increased cytochrome P450 family 24 subfamily A member 1 (CYP24A1) [65]. Mouse BMSCs treated with higher concentrations of D-galactose (20 mg/ml) showed decreased viability, increased apoptosis, impaired osteogenesis, and iron accumulation, linking D-galactose-induced aging to iron dysregulation [66]. In osteoblast lineage cells, including MC3T3-E1 and primary osteoblasts, D-galactose induced senescence, ferroptosis (as indicated by increased MDA and related markers), and oxidative stress, leading to impaired proliferation and differentiation [40, 61]. In osteoclast precursors, D-galactose promoted differentiation in RAW 264.7 cells, with the effect enhanced by insulin co-treatment [21]. Mechanistic studies have implicated pathways such as mechanistic target of rapamycin complex 1 (mTORC1) activation and heterochromatin reorganization [44], while protective effects were demonstrated using astragalus-containing serum [65] and 1,25(OH)_2_D_3_ [61]. The characteristics and applications of D-galactose-induced osteoporotic models reported across these studies are summarized in Table 5.

### Bone densitometric changes

Significant reductions in BMD were consistently observed in rodent models following D-galactose administration. Decreased BMD was reported in male ICR mice [61, 62], male Kunming mice [42], male C57BL/6 mice [37, 40, 54, 55], and female Kunming mice [56, 57]. Similarly, in rat models, diminished BMD was documented in male SD rats [30, 44, 63, 64], male Wistar rats [20, 32, 39, 47, 48, 60], male albino rats [33], and female SD rats [46]. These reductions were observed under the applied treatment conditions [45, 49, 51, 52]. While bone mineral content was not always explicitly measured, concomitant decreases in the bone content of calcium, phosphorus, and other minerals were frequently reported, indicating a generalized reduction in bone mineralization [33, 49, 58, 60].

**Table 6:** Documented skeletal alterations in rodent models induced by D-galactose

**Category of change**	**Specific change observed**	**Supported research**
Bone mass and density	↓ BMD	[19, 31–33, 40, 46–49, 54, 55, 57, 60–64]
Trabecular microarchitecture	↓ BV/TV	[21, 22, 31, 34, 36, 38, 40, 42, 44, 46, 52, 54, 60–63];
Cortical bone structure	↓ Tb.N	[21, 31–33, 37, 38, 40, 44, 51, 53, 54, 57, 60, 62, 63]
	↓ Tb.Th	[20–22, 29–33, 37–39, 44, 53, 55–57, 61–64]
	↑ Tb.Sp	[22, 31, 32, 37, 38, 41, 42, 44, 46–48, 51, 53, 54, 56, 61–64]
	↑ SMI, ↑ porosity, loss of connectivity	[31, 42, 52, 54, 58, 61, 62]
	↓ Ct.Th	[38, 41, 64]
	↑ Marrow space and adipocytes	[47, 48, 54]
Histological defects	Microfractures, resorption cavities, eroded surfaces	[29, 30, 32, 48, 56, 58]
Bone mechanical properties	↓ Maximum / ultimate load	[31, 38, 44, 46, 49, 54, 63]
	↓ Stiffness	[21, 22, 37, 38, 44, 46, 54]
	↓ Elastic modulus / strength	[31, 38, 44, 49, 64]

**Abbreviations:** ↑: increase; ↓: decrease; BMD: bone mineral density; BV/TV: bone volume/tissue volume: Ct.Th: cortical thickness; SMI: structure model index: Tb.N: trabecular number: Tb.Sp: trabecular separation: Tb.Th: trabecular thickness.

### Bone microstructural changes

A marked deterioration of bone microarchitecture was consistently induced by D-galactose treatment. This deterioration was characterized by a significant reduction in BV/TV in both mice [37, 40, 42, 54, 61, 62] and rats [20, 32, 44, 46, 47, 60, 63, 64]. The deterioration was further quantified by decreases in Tb.N [41, 42, 46, 61, 63, 64] and Tb.Th [20, 32, 44, 64], alongside an increase in Tb.Sp [41, 46, 60, 61, 63, 64]. A shift from plate-like to rod-like trabecular structures, indicated by an increased SMI, was also commonly observed [42, 54, 61]. A decrease in Conn.D was reported, reflecting a loss of trabecular connections [42, 44]. Sexual dimorphism was noted, with one study showing bone loss in males but not females among SD rats [45], while other studies demonstrated that D-galactose could induce microstructural deterioration in female rodents, particularly when combined with OVX [46, 53]. Alveolar bone loss was also exacerbated in periodontitis models [34, 43].

### Bone mechanical changes

Bone mechanical changes are defined as alterations in the ability of bone to withstand forces and deform without fracturing. Maximum load refers to the greatest force a bone can endure before fracture, while maximum stress denotes the highest internal force per unit area within the bone at the point of failure. Stiffness refers to the bone’s resistance to deformation under an applied load. Toughness indicates the energy a bone can absorb before fracture. Ultimate displacement is the maximum deformation a bone can undergo before failure, and bending strength refers to the ability of bone to resist bending forces without fracturing. Elastic modulus is a measure of bone stiffness, calculated as stress divided by strain in the elastic deformation region [67].

In three studies utilizing a dosage of 150 mg/kg/day, reductions in parameters such as maximum load, maximum stress, femoral stiffness, and break load were reported in both eight-week and 12-week experiments [31, 38, 54]. In three studies with a dosage of 100 mg/kg/day and induction periods ranging from 60 days to 20 weeks, consistent impairments were observed: reduced rigidity coefficient and maximum load in the 20-week study [44, 49, 63], decreased energy, power, and stiffness in the 10-week study [37], and diminished maximum load, stiffness, maximum strength, break strength, and elastic modulus in the 60-day study [46]. Overall, the detrimental effects of D-galactose on bone density, microstructure, and strength were consistently reported, as summarized in Table 6.

### Bone cellular changes

Bone remodeling is a continuous process involving two primary cell types: osteoclasts and osteoblasts. Osteoclasts, derived from hematopoietic stem cells, resorb old or damaged bone by secreting acids and enzymes that degrade mineralized bone [68, 69]. Osteoblasts, originating from mesenchymal stem cells, synthesize new bone by producing and mineralizing the bone matrix [69, 70].

In eight studies utilizing dosages of 200 mg/kg/day [19, 29, 30, 33, 35, 47, 48, 55], significant cellular alterations were observed. A 60-day study reported marked cavity formation, characterized by numerous large multinucleated osteoclasts at the surface of bony trabeculae, along with giant multinucleated osteoclasts on the bone plates exhibiting irregular edges [30]. An eight-week study at the same dosage revealed an increase in osteoclasts and a decrease in osteoblasts in femoral trabecular bone [33]. Additionally, two further eight-week studies identified multinucleated osteoclasts with an acidophilic cytoplasm in femoral tissue [47, 48].

At alternative dosages, cellular changes were reported sporadically across various induction periods. For example, a study with 200 mg/kg administered for eight weeks (dosage schedule not specified) [40] and another with 150 mg/kg/day for 12 weeks [62] both documented increased osteoblast apoptosis and a higher number of blue-stained senescence-associated β-galactosidase (SA-β-gal)-positive cells.

### Bone mineral changes

D-galactose treatment disrupted bone mineral homeostasis. Consistent reductions in bone content of calcium [49, 50, 58, 64], phosphorus [33, 58], and magnesium [49, 50] were noted. Serum levels of these minerals were also affected; for instance, serum magnesium levels decreased [51], while serum calcium increased in one study [33]. The organic matrix was negatively impacted, evidenced by a decrease in bone hydroxyproline content, indicating diminished collagen deposition [49, 50, 60]. An increase in bone ALP activity was frequently observed, suggesting heightened bone turnover [49, 58, 60]. D-galactose-induced alterations in bone cell activity and mineralization are summarized in Table 7.

**Table 7:** Histomorphometric changes in bone cell activity induced by D-galactose models

**Category of change**	**Specific change observed**	**Studies**
Impaired bone formation and osteoblast function	↓ BFR/BS or BFR/TV	[53, 61–63]
	↓ MAR	[61–63]
	↓ osteoblast number and differentiation	[33, 43]
	impaired mineralised nodule formation	[43, 61, 62]
	↓ collagen deposition	[40]
	↑ SA-β-gal^+^ cells (senescence)	[37, 40, 61, 62]
	impaired osteoblastogenesis and matrix maturation	[20]
Enhanced bone resorption and osteoclast activity	↑ Oc.N and surface	[30, 33, 43, 47, 48, 53, 63, 64]
	presence of active resorption cavities	[30, 47, 48]
Cellular damage and apoptosis	↑ apoptosis in bone cells	[40, 43, 61, 62]
	↓ cell viability and proliferation	[41, 61, 62]
	cytoskeleton damage	[40, 59]
	disrupted mitochondrial function	[41]
	↑ lysosomal activity	[59]
Altered systemic mineral homeostasis	↑ serum Ca (hypercalcaemia)	[33, 47–49, 58]
	↓ serum Pi	[33, 47–49]
	↑ serum Pi	[51]
	↓ serum Mg	[51, 58]
	↓ bone tissue Ca and Mg	[20, 49, 50, 64]
Altered bone matrix composition	↓ mineral-to-matrix ratio	[38]
	changes in acid phosphate substitution	[38]
No significant change (NS)	no change in labelled surfaces, MAR, or BFR	[64]
	no change in bone P or Zn content	[50]
	no change in serum Ca	[51]

**Abbreviations:** ↑: increase; ↓: decrease; BFR/BS: bone formation rate/bone surface; BFR/TV: bone formation rate/tissue volume; Ca: calcium; MAR: mineral apposition rate; Mg: magnesium; NS: no significant change: Oc.N: osteoclast number; P: phosphorus; Pi: inorganic phosphate; SA-β-gal: senescence-associated beta-galactosidase; Zn: zinc.

### Bone remodeling markers

Bone remodeling markers reflect bone cell activity and are broadly categorized into two types: formation markers and resorption markers. Formation markers, such as ALP, OCN, and P1NP, indicate osteoblast activity and new bone matrix synthesis. Resorption markers, including TRAP, CTX, cathepsin K (CTSK), and hydroxyproline, reflect osteoclast-mediated bone degradation.

**Table 8:** Alterations in bone remodeling biomarkers and signaling pathways

**Category of change**	**Specific change observed**	**Studies**
Bone remodelling biomarkers	Increased bone resorption markers (↑CTX-I, ↑ TRAP, ↑ Cathepsin K, ↑ Hyp)	[30, 37, 38, 47]
	Altered bone formation markers (↑ BALP, ↑ OCN in some contexts; ↓ P1NP, ↓ OCN, ↓ ALP in others)	[20, 37, 47, 60]
Oxidative stress parameters	Increased lipid peroxidation (↑ MDA, 4-HNE)	[19, 21, 31, 32, 38, 43, 47–50, 52, 56, 61]
	Decreased antioxidant defence (↓ SOD, ↓ CAT, ↓ GPx, ↓ GSH/GSSG, ↓ TAC/TAOC)	[31, 32, 35, 38, 47–50, 61, 62]
	Increased oxidative DNA damage (↑ 8-OHdG)	[38, 52]
Inflammatory signaling	Upregulated pro-inflammatory cytokines (↑ TNF-α, ↑ IL-6, ↑ IL-1β)	[33, 35, 38, 41, 54, 61, 66]
	Activation of NF-κB signaling	[19, 20, 38]
Cellular senescence and apoptosis	Activation of senescence pathways (↑ p16, ↑ p21, ↑ p53)	[35, 37, 40, 41, 61]
	Induction of apoptosis (↑ Bax/Bcl-2 ratio, ↑ Caspase-3)	[54]
	↓ SIRT6	[38]
Impaired osteogenic signaling	Downregulation of osteogenic genes (↓ Runx2, ↓ Col1a1, ↓ Osx)	[20, 41]
	Dysregulation of key signaling pathways (↓ SIRT1, ↓ Nrf2/HO-1, ↓ FoxO1, ↑ mTORC1)	[20, 35, 41, 44, 60, 61]
Systemic and Tissue-Level Changes	Cognitive impairment and neurodegeneration	[31, 34, 35, 37, 38]
	altered gut microbiota and barrier function	[24, 34, 38, 66]
	metabolic dysregulation (insulin resistance, dyslipidaemia)	[21]
	Mineral homeostasis disruption (↑ urinary Ca, ↓ bone dry weight, ↓ hydroxyproline)	[50, 57]
	Generalised ageing phenotypes (Hair greying/loss, reduced activity, organ damage)	[41, 54, 66]
Signaling pathway dysregulation	Activation of pro-resorptive pathways (NF-κB, ERK, mTORC1)	[19, 20, 38, 48]
	Inhibition of protective pathways (↓ Nrf2 and downstream targets HO-1, NQO1), ferroptosis	[61, 62]
	Dysregulation of Vitamin D metabolism (↓ CYP27B1, ↑ CYP24A1)	[65]
	Promotion of osteoclastogenesis (↑ RANKL, ↑ RANK/RANKL ratio, ↓ OPG, ↓ OPG/RANKL ratio)	[19, 20, 30, 33, 47, 48, 55]

**Abbreviations:** ↑: increase; ↓: decrease; 4-HNE: 4-hydroxynonenal; 8-OHdG: 8-hydroxy-2′-deoxyguanosine; ALP: alkaline phosphatase; BALP: bone-specific alkaline phosphatase; Bax: Bcl-2-associated X protein; Bcl-2: B-cell lymphoma 2; Ca: calcium; CAT: catalase; Caspase-3: cysteine-aspartic acid protease-3; Col1a1: collagen type I alpha 1 chain; CTX-I: C-terminal telopeptide of type I collagen; CYP24A1: cytochrome P450 family 24 subfamily A member 1; CYP27B1: cytochrome P450 family 27 subfamily B member 1; ERK: extracellular signal-regulated kinase; FoxO1: forkhead box O1; GPx: glutathione peroxidase; GSH/GSSG: reduced glutathione/oxidized glutathione ratio; HO-1: heme oxygenase 1; IL-1β: interleukin 1 beta; IL-6: interleukin 6; MDA: malondialdehyde; mTORC1: mechanistic target of rapamycin complex 1; NF-κB: nuclear factor kappa B; NQO1: NAD(P)H quinone oxidoreductase 1; OCN: osteocalcin; OPG: osteoprotegerin; Osx: osterix; p16: cyclin-dependent kinase inhibitor 2A; p21: cyclin-dependent kinase inhibitor 1A; p53: tumor protein p53; P1NP: procollagen type I N-terminal propeptide; RANK: receptor activator of nuclear factor κB; RANKL: receptor activator of nuclear factor κB ligand; Runx2: runt-related transcription factor 2; SIRT1: sirtuin 1; SIRT6: sirtuin 6; SOD: superoxide dismutase; TAC: total antioxidant capacity; TAOC: total antioxidant capacity; TNF-α: tumor necrosis factor alpha; TRAP: tartrate-resistant acid phosphatase.

Three studies with a dosage of 200 mg/kg/day over eight weeks reported increased bone ALP [33, 47, 48]. Two of these studies also noted elevated bone OCN levels [47, 48], suggesting an early high-turnover state. In contrast, two studies with a dosage of 150 mg/kg/day over 10 and 12 weeks reported decreased serum ALP [20, 62], with the 12-week study additionally reporting an increase in TRAP [62]. Among studies with a dosage of 120 mg/kg/day, two with eight-week induction periods reported increased serum ALP [32, 58], whereas a three-month study reported increased CTX-I and CTSK alongside reduced P1NP [38]. In the 100 mg/kg/day studies, a 20-week study reported a reduction in TRAP [63], a 10-week study documented decreased P1NP and increased CTX [37], and an eight-week study reported increased ALP [59]. Variability in the direction of change in ALP (both total and bone-specific) likely reflects different phases of osteoporosis, with early stages characterized by high bone turnover and elevated formation and resorption markers, while later stages are marked by osteoblast senescence and suppressed bone formation despite ongoing resorption.

The heterogeneity in changes in bone remodeling markers following D-galactose induction, such as the varying directions of ALP and OCN, may indicate different phases of osteoporosis. The early phase may be characterized by concurrent increases in bone formation and resorption markers, indicative of a high-turnover state, whereas the late phase may present decreased bone formation markers alongside sustained or increased bone resorption markers, indicative of compensatory failure and established osteoporosis.

### Inflammation changes

Inflammatory changes in bone induced by D-galactose administration have been documented across multiple studies. Serum and bone levels of pro-inflammatory cytokines, including tumor necrosis factor alpha (TNF-α), interleukin (IL)-6, and IL-1β, were elevated in D-galactose-treated rodents [20–22, 24, 33, 34, 43]. Additionally, enhanced pro-inflammatory Th17 responses were reported [37]. These inflammatory alterations were often accompanied by increased oxidative stress and bone resorption activity. In some models, inflammation was associated with cellular senescence and the senescence-associated secretory phenotype (SASP) [61]. The upregulation of these inflammatory markers correlated with deteriorated bone microarchitecture and loss of bone mass.

### Changes in signaling pathways

The impact of D-galactose on bone was mediated through the dysregulation of several critical signaling pathways. An imbalance in the RANKL/RANK/osteoprotegerin (OPG) pathway was consistently reported, characterized by increased RANKL expression and a decreased OPG/RANKL ratio, promoting osteoclastogenesis [19, 20, 30, 33, 47, 48, 55].

Substantial dysregulation of oxidative stress-related pathways was observed. The Nrf2 antioxidant pathway and its downstream targets, including heme oxygenase-1 (HO-1) and NAD(P)H quinone oxidoreductase 1 (NQO1), were found to be suppressed. This suppression correlated with increased markers of lipid peroxidation, such as MDA and 4-hydroxynonenal (4-HNE), as well as diminished antioxidant defenses, including SOD, CAT, glutathione peroxidase (GPx), and the glutathione ratio (GSH/GSSG) [31, 32, 35, 38, 47–50, 61, 62]. This oxidative environment was linked to the activation of pro-resorptive pathways, including nuclear factor kappa B (NF-κB) and extracellular signal-regulated kinase (ERK) signaling [19, 20, 38, 48]. Furthermore, the promotion of ferroptosis, a process driven by lipid peroxidation, was also identified [61].

Pathways governing cellular senescence and apoptosis were activated, with increases in the expression of senescence markers p16, p21, and p53 documented [35, 37, 40, 41, 61], alongside a reduction in sirtuin 6 (SIRT6) [38]. Apoptosis in bone cells was induced, as evidenced by an increased Bcl-2-associated X protein (Bax)/B-cell lymphoma 2 (Bcl-2) ratio and elevated cleaved caspase-3 levels [54]. Additionally, the mTORC1 pathway, associated with cellular senescence and inhibited autophagy, was activated [44].

Osteogenic signaling was significantly impaired, evidenced by downregulation of key osteoblast differentiation genes, including runt-related transcription factor 2 (Runx2), collagen type I alpha 1 chain (Col1a1), and Osterix [20, 41]. Dysregulation of vitamin D metabolism was also noted, with decreased expression of the activating enzyme CYP27B1 and increased expression of the catabolizing enzyme CYP24A1 [65]. The protective pathways sirtuin 1 (SIRT1) and forkhead box O1 (FoxO1) were found to be inhibited [20, 60].

The observed changes in bone remodeling biomarkers and associated signaling pathways, elucidating potential mechanisms underlying D-galactose-induced bone deterioration, are summarized in Table 8. The proposed mechanisms of D-galactose-induced bone alterations are illustrated in Figure 2.

**Figure 2.**
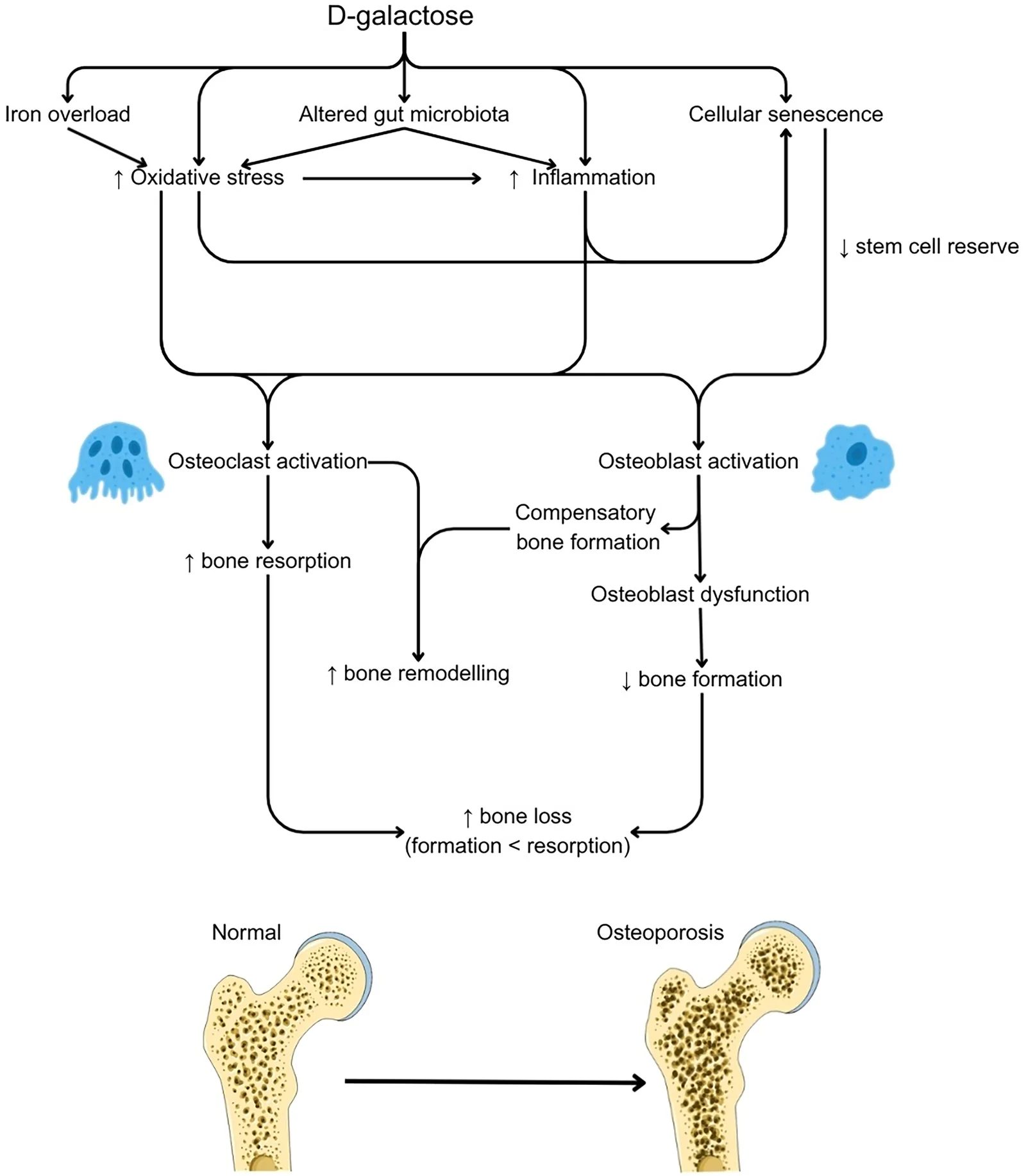
**Proposed mechanisms of D-galactose-induced bone alterations.** Schematic overview of the pathways through which D-galactose promotes bone loss and osteoporosis. D-galactose induces oxidative stress, inflammation, altered gut microbiota, iron overload, and cellular senescence, accompanied by depletion of the stem cell reserve. These interrelated processes stimulate osteoclast activation and bone resorption, while simultaneously impairing osteoblast function. Although compensatory bone formation may initially occur, persistent osteoblast dysfunction ultimately reduces bone formation. The resulting imbalance in bone remodelling, where bone formation is lower than bone resorption, leads to progressive bone loss and the development of an osteoporotic phenotype. Arrows indicate proposed mechanistic relationships between upstream ageing-related disturbances and downstream skeletal consequences. ↑ indicates an increase; ↓ indicates a decrease.

### Other changes

Other notable changes related to bone include an increased accumulation of bone marrow adipocytes [54], elevated levels of advanced glycation end products (AGEs) [21, 22], and the induction of ferroptosis in osteoblasts, characterized by lipid peroxidation and mitochondrial damage [61]. Alterations in gut microbiota were associated with changes in bone metabolism in certain models [34, 38].

## Discussion

This scoping review comprehensively summarizes evidence from 44 studies examining the effects of D-galactose on bone loss in rodent models. The findings demonstrate that D-galactose administration consistently induces osteoporotic changes across various animal strains, sexes, and ages; however, the extent and characteristics of bone loss vary according to dosage, duration, route of administration, and model-specific factors. A decision tree based on the literature to aid the readers in selecting the relevant models for various purposes (Figure 3). We propose a low-dose, extended study period (approximately 100 mg/kg/day for a minimum of three months) to accurately replicate the effects of skeletal aging, a medium dose and moderate study period (∼150–200 mg/kg/day for approximately two months) for rapid drug screening, and a high dose (∼500 mg/kg/day for about two months) to study severe skeletal ageing with compensatory failure.

**Figure 3.**
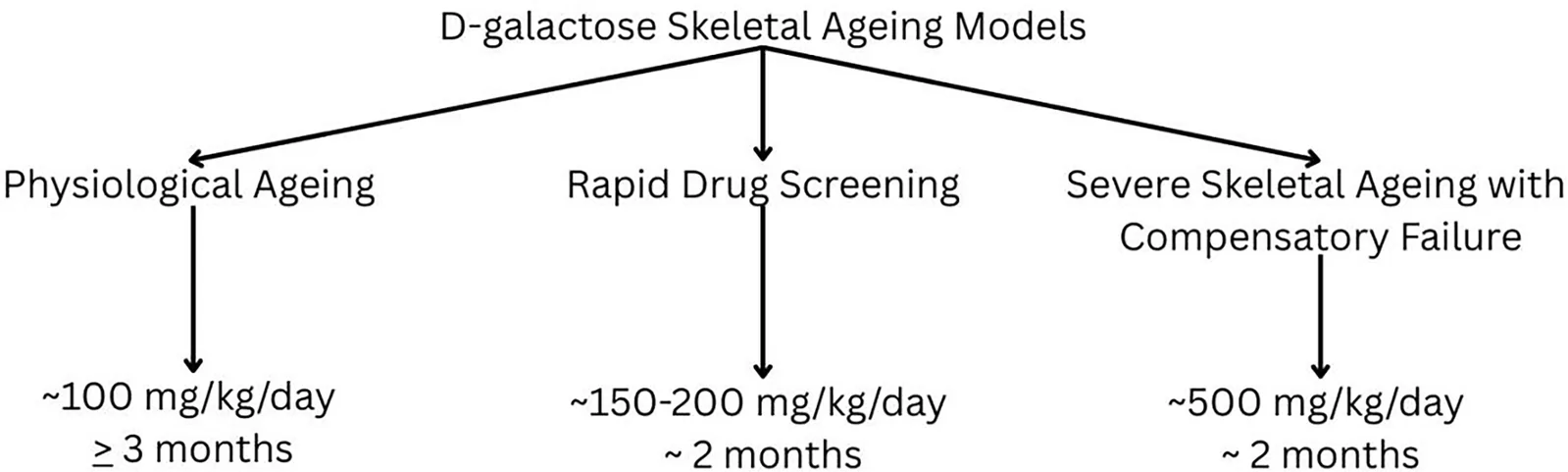
**Proposed decision tree for selecting D-galactose dose and study period according to the intended skeletal ageing phenotype.** This schematic summarises a literature-based framework to guide model selection in studies of D-galactose-induced skeletal ageing. Lower-dose, longer-duration protocols (∼100 mg/kg/day for ≥3 months) are proposed to mimic physiological skeletal ageing. Intermediate-dose, moderate-duration protocols (∼150–200 mg/kg/day for ∼2 months) are suggested for rapid drug screening, where reproducible osteoporotic changes are required within a shorter experimental window. Higher-dose protocols (∼500 mg/kg/day for ∼2 months) are proposed for modelling severe skeletal ageing with compensatory failure, characterised by more pronounced bone deterioration. The figure is intended as a practical guide for aligning D-galactose dosing strategy and study duration with the primary objective of the experimental model.

The choice between s.c. and i.p. administration in D-galactose-induced osteoporosis models significantly affects the rate and stability of skeletal deterioration. Our review suggests that s.c. administration may provide more sustained systemic exposure, potentially modeling chronic age-related bone loss more effectively. In contrast, i.p. injection may induce oxidative stress more rapidly and increase bone turnover, as observed in several studies [47, 48, 62]. However, direct comparative studies are limited, and the choice of administration route should be guided by specific research questions and outcome measures.

D-galactose treatment significantly contributes to bone loss, characterized by reduced BMD, deterioration of trabecular microarchitecture (including decreased bone volume fraction, trabecular number, and thickness, alongside increased trabecular separation), and impaired biomechanical properties [19, 23, 32, 33, 40, 41, 44, 46, 47, 49, 52, 54, 57, 60, 62, 63]. These alterations are frequently accompanied by changes in bone turnover markers, indicating disrupted bone homeostasis [37, 39, 48, 51, 60, 62, 64]. Notably, D-galactose-induced bone loss correlates with increased oxidative stress, cellular senescence, and inflammation, highlighting the role of accelerated aging in this process [20, 31, 35, 38, 40, 47, 61, 62].

The studies reviewed indicate that D-galactose may exacerbate bone loss through multiple pathways. Oxidative stress is a critical factor, as D-galactose enhances reactive oxygen species (ROS) generation and lipid peroxidation (evidenced by elevated levels of MDA and 4-HNE), while reducing the activity of antioxidant enzymes such as SOD, CAT, and GPx [19, 50, 52, 56, 62]. This oxidative environment promotes osteoblast apoptosis and inhibits osteogenic differentiation [40, 54, 62, 66]. Additionally, cellular senescence is implicated, with the upregulation of senescence markers such as p16, p21, and γ-H2A.X, as well as the SASP, contributing to impaired bone formation [37, 44, 54, 61]. Inflammatory processes also play a significant role, as D-galactose elevates pro-inflammatory cytokines, including IL-6 and TNF-α, which stimulate osteoclastogenesis and bone resorption [20, 21, 24, 47]. More recently, ferroptosis has been identified as another mechanism, with D-galactose triggering ferroptosis in osteoblasts by suppressing the Nrf2/GPX4 pathway, leading to iron accumulation and lipid peroxidation [61, 66]. Hormonal and metabolic dysregulation further contributes, as evidenced by disrupted vitamin D metabolism [61, 65], altered mineral homeostasis (e.g., reduced calcium, magnesium, and phosphorus content) [49, 50, 58], and impaired glucose metabolism via the polyol pathway [36]. However, many studies rely on observational data where increases in markers like MDA or p16 coincide with bone loss. Fewer studies utilize specific inhibitory or ‘rescue’ models (e.g., GPX4 overexpression or specific ferroptosis inhibitors) to definitively establish these pathways as primary drivers of the osteoporotic phenotype. Thus, while the D-galactose model remains a robust surrogate for age-related bone loss, the precise mechanistic hierarchy requires further validation through targeted knockout or intervention-based experimental designs.

The effects of D-galactose on bone are influenced by multiple model-specific factors. Sex differences have been noted, with male rodents generally exhibiting greater susceptibility to D-galactose-induced bone loss than females, possibly due to differential effects on gonadal function [45]. Furthermore, the protective role of endogenous estrogen on the skeletal system may contribute to these differences. Estrogen exerts a potent anti-inflammatory effect and maintains the OPG/RANKL ratio [71], which protects the skeleton against accelerated senescence induced by D-galactose. Conversely, the protective effects of estrogen in male rodents are weaker, rendering their osteoblasts more vulnerable to D-galactose-induced oxidative stress and apoptosis. Additionally, sex-specific differences in the expression of senescence markers, such as p16 and p21 [72], suggest that D-galactose may accelerate the depletion of the mesenchymal stem cell pool more rapidly in males. While female rodents ultimately experience significant bone loss, this typically requires a longer induction period or higher dosage to overcome initial hormonal protection. However, combined models, such as OVX with D-galactose administration, effectively induce bone loss in female animals [46, 53].

Age also influences outcomes, as younger rodents (6–12 weeks old) are commonly employed in studies. The severity of bone loss varies across strains, including Wistar, SD, C57BL/6, ICR, and Kunming [19–24, 29–64]. Dosage and duration are critical factors, with D-galactose doses ranging from 10–1,500 mg/kg/day, though the most frequently used dose is 200 mg/kg/day [19, 29, 30, 33, 35, 47, 48, 55]. Induction periods of 8–12 weeks are generally sufficient to induce marked bone loss, while longer durations of three to five months intensify aging-like phenotypes [49, 51, 54, 61]. The route of administration also plays a role, as both s.c. and i.p. injections are effective, while oral administration is less commonly used but has been shown to induce bone loss [36, 55].

Despite the consistency of findings, several limitations warrant acknowledgment. The lack of standardization in D-galactose dosage, administration routes, and treatment durations complicates direct comparisons among studies and hinders consensus on an optimal protocol. Furthermore, a pronounced sex and age bias exists, as most studies utilized male rodents, thereby limiting insights into the effects of D-galactose in female models, particularly in the context of postmenopausal osteoporosis. Mechanistic understanding remains incomplete; emerging knowledge about bone biology, including the gut-bone axis and ferroptosis, should be integrated into the skeletal effects of D-galactose.

Regarding the gut-bone axis, D-galactose administration disrupts gut barriers, induces inflammation, reduces microbial diversity and the Firmicutes/Bacteroidetes ratio, and alters the abundance of specific gut bacteria [24, 34]. These detrimental changes may lead to increased systemic inflammation and altered nutrient metabolism, both of which can compromise bone health. Additionally, recent evidence underscores ferroptosis as a critical mechanism that interacts with oxidative stress and cellular senescence to accelerate skeletal aging [73]. Induction by D-galactose results in the accumulation of intracellular ferrous iron and downregulation of GPX4, a key antioxidant enzyme [62, 66]. This suppression of GPX4 initiates lipid peroxidation, which directly damages osteoblast membranes and induces ferroptotic cell death.

Future research should prioritize the standardization of experimental protocols, determining optimal D-galactose dosages and administration routes tailored to specific research objectives, such as aging or postmenopausal osteoporosis. Incorporating female models, particularly in conjunction with OVX or other hormonal manipulations, is also essential. Further studies should investigate novel mechanisms, including ferroptosis, the gut microbiota, and epigenetic regulation in D-galactose-induced bone loss. Moreover, D-galactose models should be more widely employed to validate therapeutic strategies, including antioxidants, senolytics, and modulators of the gut microbiota, with particular emphasis on evaluation in aged animals.

From a translational perspective, the D-galactose model exhibits hallmark features paralleling human age-related osteoporosis, notably a shift toward a high-turnover bone loss state driven by systemic oxidative stress and chronic low-grade inflammation. Similar to human senescence, D-galactose-induced skeletal alterations are characterized by significant reductions in trabecular bone volume and disruption of the OPG/RANKL ratio. However, challenges persist in translating these preclinical findings to clinical practice. Unlike the gradual, multi-decadal progression of osteoporosis in humans, the D-galactose model represents an accelerated senescence phenotype induced by metabolic overload, potentially bypassing certain age-dependent epigenetic modifications observed in humans. Additionally, rodents lack the Haversian remodeling system found in human cortical bone [74], indicating that cortical thinning in D-galactose models may not accurately replicate the porosity observed in elderly patients. While the model effectively screens antioxidants and bone-protective agents, caution is warranted when extrapolating specific dosages and timelines to human clinical trials.

This review also has several limitations. It considered only articles published in English and Mandarin and indexed in selected databases, which may have introduced language and publication bias. Significant heterogeneity across the included studies precluded meta-analysis, rendering the synthesis descriptive rather than quantitative. Furthermore, relevant unpublished findings or studies from less accessible sources may have been overlooked. Finally, the review’s focus on preclinical rodent models limits integration with human or clinical evidence, restricting the direct applicability of the conclusions to clinical osteoporosis.

## Conclusion

In conclusion, D-galactose-induced osteoporosis in rodents replicates several hallmarks of age-related bone loss, including reduced bone mass and density, microarchitectural deterioration, altered remodeling, mineral deficits, and molecular perturbations. The model operates through mechanisms such as oxidative stress, cellular senescence, inflammation, and ferroptosis, and is versatile and reproducible across different strains and sexes, making it a valuable platform for studying osteoporosis and screening potential interventions. However, the current evidence base is constrained by a relatively small number of studies, heterogeneity in dosages, induction periods, and assessment methods, as well as a lack of standardization across experimental protocols. Future research should involve larger cohorts, adopt standardized models, and incorporate long-term evaluations to clarify dose-response relationships, deepen mechanistic insights, and enhance the translational relevance of D-galactose models for investigating interventions targeting senile osteoporosis.

## Data Availability

No raw data were generated for this study.
